# Conifer Needle Phyllosphere as a Potential Passive Monitor of Bioaerosolised Antibiotic Resistance Genes

**DOI:** 10.3390/antibiotics11070907

**Published:** 2022-07-07

**Authors:** Paul B. L. George, Samantha Leclerc, Nathalie Turgeon, Marc Veillette, Caroline Duchaine

**Affiliations:** 1 Département de Médecine Moléculaire, Université Laval, Quebec City, QC G1V 0A6, Canada; 2 Département de Biochimie, de Microbiologie et de Bio-Informatique, Université Laval, Quebec City, QC G1V 0A6, Canada; samantha.leclerc.3@ulaval.ca (S.L.); caroline.duchaine@bcm.ulaval.ca (C.D.); 3 Centre de Recherche de l’Institut Universitaire de Cardiologie et de Pneumologie de Québec, Quebec City, QC G1V 4G5, Canada; nathalie.turgeon@criucpq.ulaval.ca (N.T.); marc.veillette@criucpq.ulaval.ca (M.V.)

**Keywords:** biomonitoring, phyllosphere, resistome, swine production, bioaerosols

## Abstract

Monitoring antibiotic resistance genes (ARGs) is vital to the One Health approach to tackling the antibiotic resistance crisis. It has been suggested that conifer needles can be used as passive bioaerosol samplers. Here, the use of conifer needles as biomonitors of ARGs in bioaerosols was assessed as a proof-of-concept. Needles were collected from trees surrounding pig farms, villages, and forest sites in Québec, Canada. Needles were homogenised and DNA was extracted. Results of qPCR analyses showed biomass estimates were consistent across samples. Number and quantity of ARGs was significantly lower in forest sites when compared to the farm and village, comprising a distinct resistome. Consistent with previous findings, the most common ARGs were tetracyclines and sulfonamides, which were found close to agricultural activities. Although results were limited, there is great potential for using the conifer phyllosphere as a passive bioaerosol sampler. This method represents an accessible way to promote ARG surveillance over long distances from point sources.

## 1. Introduction

The One Health concept posits that the health of people, animals, and the environment are interlinked such that multidisciplinary efforts are needed at all scales to ensure global well-being [1]. In this context, the growing crisis of antibacterial resistance can be thought of as the ultimate One Health initiative [2]. When antimicrobial resistant bacteria (ARB) enter the wider environment, they can transfer antibiotic resistance genes (ARGs) to naïve organisms via horizontal gene transfer, leading to a proliferation of antibiotic resistance [3]. Antibiotic resistance may cause 10 million deaths globally by 2050, assuming a worst-case scenario [2]. Monitoring the spread of ARGs out of farming operations is thus an important component of a One Health approach to assessing antibiotic resistance. 

Global estimates suggest swine consume 172 mg kg^−1^ of antimicrobial products [4]. A concerning increase in both ARB and ARGs has been reported in swine production, where they are commonly identified in faeces [5], including multiple reports of the *mcr*-1 gene, which confers resistance against colistin, an important antibiotic of last-resort [6,7,8]. Swine faeces are also a source of bioaerosols, which can disseminate ARB and ARGs over long distances [9,10,11]. However, bioaerosol monitoring is often limited to temporally and geographically constrained point measurements [12]. 

Passive bioaerosol collectors have great utility as they can collect aggregate samples of long periods of time [13]. Galès et al. [12] demonstrated that pine needles trapped airborne bacteria emitted from a composting facility and suggested their use as passive biomonitors of bioaerosols. A similar method has also been employed to assess aerial dispersal patterns of DDT [14]. Windbreaks of conifers surrounding farms contain ammonia [13], odour [15], and dust [16] emissions from livestock buildings. If conifer needles can be proven effective at trapping and recovering aerosolised ARGs they could become an accessible tool for large-scale ARG monitoring.

The utility of the conifer needle phyllosphere as a passive bioaerosol sampler was assessed. The objective was to demonstrate that ARGs can be recovered from the conifer phyllosphere and that the composition of ARGs would change in different habitats. The aim was to demonstrate that this method could be applied to the study of airborne ARG dispersal. To the authors’ knowledge, this is the first time that this methodology has been applied to the study of ARG dispersal. 

## 2. Results

**Figure 1:**
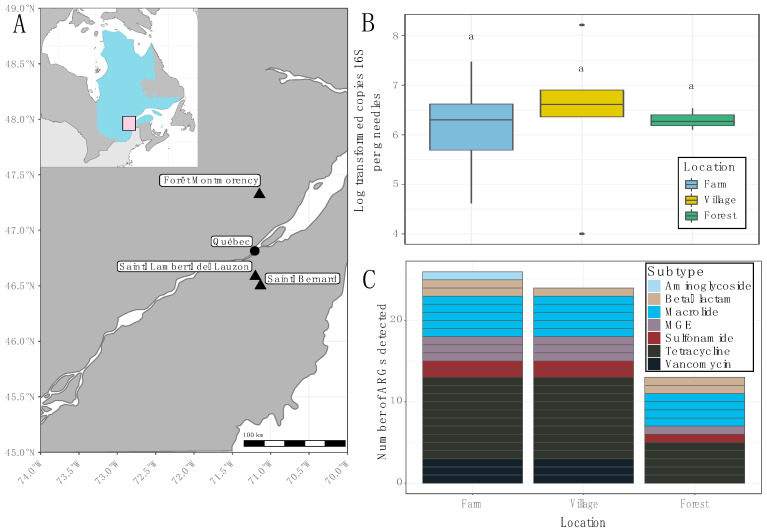
Summary information of conifer needle samples. (**A**) Sampling sites in the province of Québec, Canada; inset map shows the position of Québec within Canada. (**B**) Biomass estimates based on log-transformed copy number of 16S rRNA gene per g of conifer needles. Boxes represent the data within the first and third quartiles; the horizontal line marks medians; whiskers represent 1.5× the interquartile range. (**C**) Total number of ARGs detected via qPCR at each sampling site.

Conifer needles were collected from three locations to test their efficacy for monitoring ARGs in bioaerosols. Trees were selected immediately adjacent to farms (−3–50 m), and in villages 3–9 km distant from study farms in Saint-Bernard and Saint-Lambert-de-Lauzon, QC, Canada. Reference samples were collected from trees in the Forêt Montmorency. Sample location did not impact biomass estimates based on 16S rRNA gene copies per g needles (Figure 1A,B). However, analyses of ARGs revealed stark contrasts between sample locations. Of the 37 ARGs and 3 mobile genetic elements (MGEs) targeted by the qPCR panel, 13 were not detected. The remaining 26 were detected in the phyllosphere of trees surrounding farms, 24 were detected in needles from village sites, and only 13 were detectable in the forest site (Figure 1C). Of these, the largest subtype detected in each site was tetracyclines; macrolides were also shared across sampling areas (Figure 1C).

**Figure 2:**
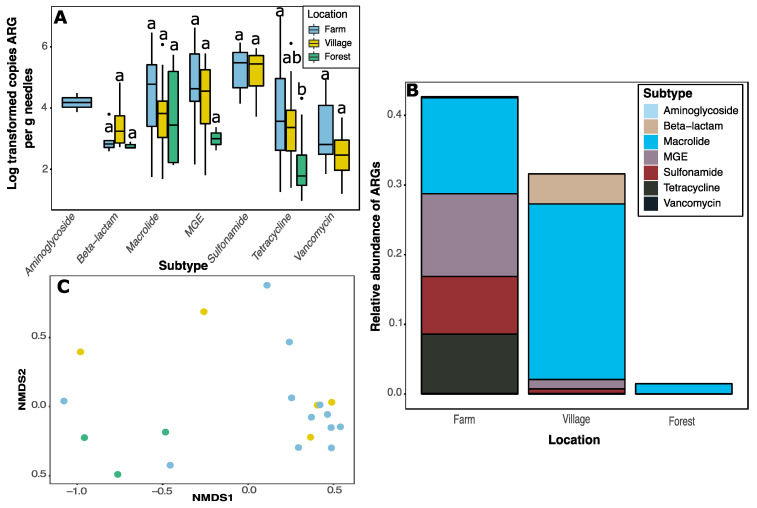
Analysis of ARGs detected in conifer needle samples. (**A**) Boxplot of copy numbers of ARG subtype. Boxes represent the data within the first and third quartiles; the horizontal line marks medians; whiskers represent 1.5× the interquartile range; black dots denote outliers. (**B**) Relative abundance of ARGs in each sampling area. (**C**) Nonmetric multidimensional scaling ordination of needle phyllosphere samples based on copy number estimates of all ARGs. Each dot indicates a single sample. Colours correspond to the legend in panel (**A**).

The marked reduction in number and proportion of ARGs detected in forest samples can be clearly seen when copy numbers were quantified (Figure 2). No sulfonamide or vancomycin ARGs were detected in forest samples; aminoglycoside ARGs were only detected in farm samples (Figure 2A). Copy numbers of tetracycline ARGs were significantly (F_2,122_ = 5.2, *p* = 0.007) lower in forest as compared to farm (*p* = 0.006) samples (Figure 2A). Due to a limited sampling campaign, evident differences in copy number of MGEs and beta-lactam genes were not significant, but likely biologically relevant. 

Only 11 of the target genes were detected in the samples from Forêt Montmorency (Figure 2B; Supplementary Material Table S1). Macrolides made up the highest proportion of ARGs in all sampling areas. They were especially prevalent in village samples. In forest samples, there were also large proportions of MGEs, sulfonamides, and tetracycline ARGs when compared to other sampling areas. Nonmetric dimensional scaling (NMDS) and permutational analysis of variance (permanova; F_2,17_ = 2.00; *p* = 0.02) revealed that forest samples cluster separately from other sites (Figure 2C). There were no differences in homogeneity of variances (F_2,17_ = 0.75, *p* = 0.49).

## 3. Discussion

These results demonstrate the potential use of conifer needles as passive bioaerosols monitors as posited by Galès et al. [12]. Due to availability, several conifer species were used in this study, demonstrating multiple species can be used in this manner. Most trees sampled were *Picea* sp., which are already recommended for windbreaks [15] as they trap particulate matter more effectively than other trees, limiting agricultural air pollution [15,16]. Since biomass estimates did not differ by sampling site, it is assumed that differences in ARG were not introduced by laboratory or handling contamination. Rather, the markedly different proportions of studied ARGs suggests that they represent a distinct resistome present in the very different environment of the Boreal forest [17]. 

Tetracylines are some of the most commonly used antibiotics in Canadian agriculture [18] as well as in swine production generally [19]. ARGs targeting these antibiotics are commonly found in swine faeces and near production facilities [5] including in bioaerosols [20,21]. Further evidence shows that tetracycline resistance genes are common in soils and waterways in proximity to swine barns [21,22,23,24]. High copy numbers of tetracycline ARGs in farm sites including *tetL* in comparison to forest samples exemplify the large footprint of tetracycline on the adjacent environment (Figure 2C). Since tetracycline resistance genes have been identified in at least 126 bacterial genera [4], this suggests distinct differences in tetracycline genes across regions.

Although the sampling effort was limited, marked differences in abundances of individual ARGs were observed. Indeed, since this work was a proof-of-concept, the ability to interpret these results is limited. Village sites were surrounded by swine production facilities and arable land. There is overwhelming evidence that such activities emit ARGs into the environment at high rates [5]. In contrast, the forest site is a protected area located well away from agricultural activities (Figure 1A). There are likely important trends in MGEs and beta-lactams that could be revealed with a more intensive sampling campaign. MGEs commonly facilitate the horizontal transfer of ARGs [25]. This may be amplified in response to stressors such as toxic particles in the exhaust of combustion engines [26] or heavy metal contamination [27]. Since exposure to both exhaust and heavy metals can occur in agricultural settings, this may explain the reduced copy number of MGEs in forest sites. This is an especially important avenue for future work in the context of sustainable rural development.

The phyllosphere represents a highly diverse and populous microbiome, which can be used to answer important questions of microbial biogeography [28]. The conifer phyllosphere was chosen because these trees keep their foliage year-round and their prevalence in the northern hemisphere as windbreak species. However, it would be interesting to see if the phyllospheres of deciduous trees could also be used in this manner. Indeed, there is some evidence, that the resistome of agricultural phyllosphere samples had distinct ARG communities compared to more natural forest sites in China [29]. Furthermore, extending sampling campaigns throughout the year could reveal seasonal trends in airborne ARG emissions.

In conclusion, the study demonstrates that the conifer needle phyllosphere can be used to monitor ARG dispersal in the environment. Since conifers are already a common feature of windbreaks on farms, implementing ARG monitoring using this method on a wider scale is an intuitive next step, adhering to One Health principles of integrating human and environmental components to addressing antibacterial resistance. This method can be easily implemented in aerosolised ARG monitoring programmes as it precludes the need for specialised air samplers and has the potential lead to meaningful dialog with land managers. The results also highlight the ability of agricultural bioaerosols to spread ARGs over long distances and how they can overshadow ambient ARG distributions. Future work should increase sampling intensity both in terms of number of sites and replicates to overcome variability in ARG presence and to better define the extent of their emission from point sources. This should also be complemented by the sampling or indoor or exhaust air with more conventional methods. Different farm types should also be investigated for farm-specific patterns in ARGs. 

## 4. Materials & Methods

In summer 2021, conifer needles were collected from trees surrounding swine barns in Saint-Bernard, QC, Canada (n = 12) as well as from trees in the village proper and the nearby village of Saint-Lambert-de-Lauzon (n = 5) between 3 to 18 km from the swine barns. Control trees were selected in Forêt Montmorency (n = 3), a 397 km^2^ protected forest ~80 km due north of the other locations (Figure 1A). Samples were collected over two excursions at farm sites to overcome any biases introduced by rain events during the first excursion. Subsequent sampling events to other locations were not possible due to limitations on site access during the pandemic. Most trees were identified as *Picea* sp.; however, there were 4 *Abies balsema* and 1 *Pinus strobus*. 

Following Galès et al. [12], 2 branch ends from each tree approximately 20 cm long at 1.5–2 m height were collected in a sterile plastic bag. After collection, 15 g of needles from each tree were plucked and homogenised in a 50 mL solution of 0.05% Tween20 saline buffer using a Stomacher Mix1 (Aes Laboratoire, Bruz, France) for 5 min. Large debris were removed by differential centrifugation, at 250 rpm for 3 min, and the supernatant was centrifuged at top speed for 30 min to create pellets. Pellets were stored at −20 °C until DNA extraction with a DNeasy^®^ PowerSoil^®^ Kit (Qiagen, Montreal, QC, Canada) per manufacturer’s instructions. Each extraction represents a single tree (n_total_ = 20).

Each sample was subjected to a qPCR panel of 37 ARGs, and 3 MGEs, and the 16S rRNA gene using a Bio-Rad CFX-384 Touch^TM^ Real-Time PCR Detection System (Bio-Rad, Hercules, CA, USA). This panel includes ARGs that confer resistance to aminoglycosides (3), beta-lactams (10), colistin (1), macrolides, (5), quinolones (2), tetracyclines (10), sulfonamides (2), and vancomycin (4). Full primer sequences can be found in Table S1. All primers used SYBR Green dye fluorescence except for those of the 16S rRNA, *mcr1*, and *blaCTX-M-1* genes, which used a FAM probe for fluorescence. The SYBR Green primers shared a thermoprotocol of an initial step of 95 °C for 3 min; then 95 °C for 10 s; 60 °C for 30 s; and 55 °C for 31 s; with a melt curve of 55 °C for 5 s + 0.5 °C/cycle. The FAM primers used a shared thermoprotocol of 95 °C for 3 min; then 95 °C for 20 s; and 62 °C for 1 min for 40 cycles. Analyses with efficiency curves between 90–110% were used.

DNA extraction blanks were included with all qPCR analyses. If reactive, the starting quantity (*Sq*) values of corresponding blanks were subtracted from the *Sq* values of samples following qPCR analyses. There were no reactions in no template qPCR negative controls. Gene copy numbers were estimated using the formula:

$$Copy\;number\;per\;g\;needles=\left(\frac{\left(\frac{Sq}{2}\right)\times 100}{7.5}\right)\times 50/Wn$$

where *Sq* is the starting quantity estimate from qPCR analyses from 2 μL of DNA, 7.5 (mL) is the volume of supernatant used to make aliquots, 50 (mL) is the total volume of starting solution, and *Wn* is the weight of needles. Copy numbers were log-transformed for all statistical analyses. Relative abundance of ARGs was calculated by dividing untransformed ARG copy number by 16S copy number.

Statistical analyses were preformed using R v. 4.0.2 [30]. Significant differences between gene copy numbers were assessed using one-way analyses of variance with Tukey’s post-hoc tests. Ordination, permanova, and homogeneity of variance tests (betadisper) were performed with the vegan package [31].

## Data Availability

Data is available upon request to the corresponding author.

## Supplementary Materials

The following supporting information can be downloaded at: https://www.mdpi.com/article/10.3390/antibiotics11070907/s1, Table S1: List of gene targets and primers used for qPCR analyses. Genes noted by * used a FAM probe, all others used SYBR Green fluorescence [32,33,34,35].
