# Supplementary material for: Conifer Needle Phyllosphere as a Potential Passive Monitor of Bioaerosolised Antibiotic Resistance Genes
Source: Antibiotics (Basel). 2022 Jul 7;11(7):907. doi: 10.3390/antibiotics11070907 (PMC9312085; doi:10.3390/antibiotics11070907)
Supplement: Supplementary file 1 [file antibiotics-11-00907-s001.zip › antibiotics-1746745-supplementary.pdf]

Supplementary Information for

Conifer Needles as Passive Monitors of Bioaerosolised Antibiotic Resistance Genes

Paul B.L. George, Samantha Leclerc, Nathalie Turgeon, Marc Veillette, Caroline Duchaine.

Table S1. List of gene targets and primers used for qPCR analyses. Genes noted by * used a FAM probe, all others used SYBR Green fluorescence. The letter ^{D} denotes whether a gene was detected at a quantifiable limit in at least one sample.

Gene	Primer Sequence	Ref.
16S *	**F:** GGTAGTCYAYGCMSTAAACG **R:** GACARCCATGCASCACCTG **P:** TKCGCGTTGCDTCGAATTAAWCCAC-BHQ	[1]
aac(6')-II	**F:** CGACCCGACTCCGAACAA **R:** CGACCCGACTCCGAACAA	[2]
aac(6')-Ib^{D}	**F:** CGTCGCCGAGCAACTTG **R:** CGGTACCTTGCCTCTCAAACC	[2]
aac(3)-iid_iii_iif_iia_iie	**F:** CGATGGTCGCGGTTGGTC **R:** TCGGCGTAGTGCAATGCG	[2]
blaCMY2	**F:** AAAGCCTCATGGGTGCATAAA **R:** ATAGCTTTTGTTTGCCAGCATCA	[2]
blaCTX-M-1,3,15 *	**F:** CGTACCGAGCCGACGTTAA **R:** CAACCCAGGAAGCAGGCA **P:** CCARCGGGCZENGCAGYTGGTGAC	[3]
blaGES	**F:** GCAATGTGCTCAACGTTCAAG **R:** GTGCCTGAGTCAATTCTTTCAAAG	[2]
blaOXA ^{D}	**F:** CGACCGAGTATGTACCTGCTTC **R:** TCAAGTCCAATACGACGAGCTA	[2]
blaMOX/blaCMY	**F:** CTATGTCAATGTGCCGAAGCA **R:** GGCTTGTCCTCTTTCGAATAGC	[2]
blaSHV-11	**F:** TTGACCGCTGGGAAACGG **R:** TCCGGTCTTATCGGCGATAAAC	[2]
blaTEM ^{D}	**F:** AGCATCTTACGGATGGCATGA **R:** TCCTCCGATCGTTGTCAGAAGT	[2]
blaVEB	**F:** CCCGATGCAAAGCGTTATG **R:** GAAAGATTCCCTTTATCTATCTCAGACAA	[2]
blaVIM	**F:** GCACTTCTCGCGGAGATTG **R:** CGACGGTGATGCGTACGTT	[2]
erm(35) ^{D}	**F:** CCTTCAGTCAGAACCGGCAA **R:** GCTGATTTGACAGTTGGTGGTG	[2]
ermB ^{D}	**F:** GAACACTAGGGTTGTTCTTGCA **R:** CTGGAACATCTGTGGTATGGC	[2]
ermF ^{D}	**F:** CAGCTTTGGTTGAACATTTACGAA **R:** AAATTCCTAAAATCACAACCGACAA	[2]

ermT [D]	**F:** GTTCACTAGCACTATTTTTAATGACAGAAGT **R:** GAAGGGTGTCTTTTTAATACAATTAACGA	[2]
ermX [D]	**F:** GCTCAGTGGTCCCCATGGT **R:** ATCCCCCCGTCAACGTT	[2]
imp-marko	**F:** GGAATAGAGTGGCTTAATTC **R:** GGTTTAACAAAACAACCACC	[2]
int1-a-marko [D]	**F:** CGAAGTCGAGGCATTTCTGTC **R:** GCCTTCCAGAAAACCGAGGA	[2]
is26 [D]	**F:** ATGGATGAAACCTACGTGAAGGTC **R:** CGGTACTTAATCTGTCGGTGTTCA	[2]
mcr-1 *	**F:** CACATCGACGGCGTATTCTG **R:** CAACGAGCATACCGACATCG	[4]
qepA	**F:** GGGCATCGCGCTGTTC **R:** GCGCATCGGTGAAGCC **P:** CTACAGACCZENGACCAAGCCGA	[2]
qnrB	**F:** TCACCACCCGCACCTG **R:** GGATATCTAAATCGCCCAGTTCC	[2]
sul1 [D]	**F:** GCCGATGAGATCAGACGTATTG **R:** CGCATAGCGCTGGGTTTC	[2]
sul2 [D]	**F:** TCATCTGCCAAACTCGTCGTTA **R:** GTCAAAGAACGCCGCAATGT	[2]
tet32 [D]	**F:** CCATTACTTCGGACAACGGTAGA **R:** CAATCTCTGTGAGGGCATTTAACA	[2]
tetA [D]	**F:** CTCACCAGCCTGACCTCGAT **R:** CACGTTGTTATAGAAGCCGCATAG	[2]
tetC [D]	**F:** ACTGGTAAGGTAAACGCCATTGTC **R:** ATGCATAAACCAGCCATTGAGTAAG	[2]
tetL [D]	**F:** ATGGTTGTAGTTGCGCGCTATAT **R:** ATCGCTGGACCGACTCCTT	[2]
tetM [D]	**F:** GGAGCGATTACAGAATTAGGAAGC **R:** TCCATATGTCCTGGCGTGTC	[2]
tetO [D]	**F:** CAACATTAACGGAAAGTTTATTGTATACCA **R:** TTGACGCTCCAAATTCATTGTATC	[2]
tetQ [D]	**F:** CGCCTCAGAAGTAAGTTCATACACTAAG **R:** TCGTTCATGCGGATATTATCAGAAT	[2]
tetS [D]	**F:** TTAAGGACAAACTTTCTGACGACATC **R:** TGTCTCCCATTGTTCTGGTTCA	[2]
tetW [D]	**F:** ATGAACATTCCCACCGTTATCTTT **R:** ATATCGGCGGAGAGCTTATCC	[2]
tetX [D]	**F:** AAATTTGTTACCGACACGGAAGTT **R:** CATAGCTGAAAAAATCCAGGACAGTT	[2]
tnpA [D]	**F:** AATTGATGCGGACGGCTTAA **R:** TCACCAAACTGTTTATGGAGTCGTT	[2]
vanA [D]	**F:** GGGCTGTGAGGTCGGTTG **R:** TTCAGTACAATGCGGCCGTTA	[2]

vanB [D]	**F:** TTGTCGGCGAAGTGGATCA **R:** AGCCTTTTTCCGGCTCGTT	[2]
vanRA [D]	**F:** CCCTTACTCCCACCGAGTTTT **R:** TTCGTCGCCCCATATCTCAT	[2]
vanSA	**F:** CGCGTCATGCTTTCAAAATTC **R:** TCCGCAGAAAGCTCAATTTGTT	[2]

Note: F indicates forward primer sequences; R indicates reverse primer sequences; P indicates FAM probe sequences

References

(1) Bach, H.-J.; Tomanova, J.; Schloter, M.; Munch, J. C. Enumeration of Total Bacteria and Bacteria with Genes for Proteolytic Activity in Pure Cultures and in Environmental Samples by Quantitative PCR Mediated Amplification. *Journal of Microbiological Methods* **2002**, *49* (3), 235–245. https://doi.org/10.1016/S0167-7012(01)00370-0.

(2) Stedtfeld, R. D.; Guo, X.; Stedtfeld, T. M.; Sheng, H.; Williams, M. R.; Hauschild, K.; Gunturu, S.; Tift, L.; Wang, F.; Howe, A.; Chai, B.; Yin, D.; Cole, J. R.; Tiedje, J. M.; Hashsham, S. A. Primer Set 2.0 for Highly Parallel QPCR Array Targeting Antibiotic Resistance Genes and Mobile Genetic Elements. *FEMS Microbiology Ecology* **2018**, *94* (9), fiy130. https://doi.org/10.1093/femsec/fiy130.

(3) Roschanski, N.; Fischer, J.; Guerra, B.; Roesler, U. Development of a Multiplex Real-Time PCR for the Rapid Detection of the Predominant Beta-Lactamase Genes CTX-M, SHV, TEM and CIT-Type AmpCs in Enterobacteriaceae. *PLoS ONE* **2014**, *9* (7), e100956. https://doi.org/10.1371/journal.pone.0100956.

(4) Nijuis, R.H.T.; Veldman, K.T.; Schelfaut, J.; Van Essen-Zandbergen, A.; Wessels, E.; Claas, E.C.J.; Gooskens, J. Detection of the Plasmid-Mediated Colistin-Resistance Gene Mcr-1 in Clinical Isolates and Stool Specimens Obtained from Hospitalized Patients Using a Newly Developed Real-Time PCR Assay. *J. Antimicrob. Chemother.* **2016**, *71*, 2344-2346.
